# Knowledge of obstetric danger signs and its associated factors among pregnant women in Angolela Tera District, Northern Ethiopia

**DOI:** 10.1186/s13104-019-4639-8

**Published:** 2019-09-23

**Authors:** Gashaw Garedew Woldeamanuel, Gorems Lemma, Betregiorgis Zegeye

**Affiliations:** 1 Department of Biomedical Sciences, School of Medicine, College of Medicine and Health Sciences, Wolkite University, P.O. Box 07, Wolkite, Ethiopia; 2 Department of Public Health, College of Health Sciences, Debre Berhan University, Debre Berhan, Ethiopia

**Keywords:** Knowledge, Obstetric danger signs, Pregnant women, Angolela Tera district

## Abstract

**Objective:**

Knowledge of danger signs of obstetric complications is the first essential step in accepting appropriate and timely referral to maternal and newborn care units. The objective of this study was to assess the knowledge of obstetric danger signs and associated factors among pregnant women in Angolela Tera district, Northern Ethiopia. A community based cross sectional study was conducted among 563 pregnant women who were selected by multi-stage cluster sampling technique. Data were collected using a pre-tested and interviewer administered structured questionnaire. Descriptive statistics and binary logistic regression analysis were performed using statistical package for social sciences (SPSS) version 23.

**Results:**

A total of 563 pregnant women were included in the study. About 211 (37.5%) women were knowledgeable about obstetric danger signs. Attending formal education, urban residence, time taken less than 20 min to reach health facility on foot, two or more history of pregnancies and receiving health education were found to be significantly associated with being knowledgeable about obstetric danger signs. A significant proportion of pregnant women in the study area were not knowledgeable about obstetric danger signs. Hence, continuous health education and appropriate counseling to pregnant mothers should be performed.

## Introduction

Globally, an estimated 10.7 million mothers died from 1990 to 2015 due to obstetric complications and maternal mortality remains high in developing countries [1]. In 2015, approximately 99% of the global maternal deaths occurred in developing countries with majority of these deaths occurred in sub-Saharan Africa where the majority of women lack knowledge about obstetric danger signs [1–3]. About 73% of all maternal deaths were due to direct obstetric causes such as severe hemorrhage, pregnancy induced hypertension, unsafe abortion, complications from delivery and maternal infections [2–4].

Knowledge of the danger signs of obstetric complications is one aspect of obstetric problem recognized at the individual, family and community level [5, 6]. The danger signs could occur during pregnancy (vaginal bleeding, swollen hands/face, decreasing fetal movement and blurred vision), delivery (vaginal bleeding, prolonged labor, convulsions and retained placenta) and post partum period (vaginal bleeding, loss of consciousness and fever) [5, 7]. While most women have uneventful pregnancies and childbirth, all pregnancies are at risk and around 15% of pregnant women will develop a potentially life-threatening obstetrics complication that requires obstetrical interventions to survive [8].

Knowledge of the danger signs of obstetric complications is essential for early recognition of the problem, appropriate and timely referral to obstetric care [5, 9]. This will help to achieve the target of reducing global maternal mortality to less than 70 maternal deaths per 100,000 live births by 2030 [1, 10]. However, previous research conducted in developing countries showed a low status of women knowledge on danger signs during pregnancy, delivery and postpartum period. For instance, study done in rural Uganda showed that only 19% of women had knowledge of three or more key danger signs during pregnancy, delivery and post-partum period [11]. Moreover, studies conducted in Somali region of Ethiopia [12] and Tanzania [13] showed that 15.5% and 31.3% of women were knowledgeable for obstetric danger signs, respectively.

Although raising women’s knowledge of obstetric danger signs is important to reduce maternal deaths, there are limited studies in the northern Ethiopia that assessed knowledge of obstetric danger signs and associated factors. This study therefore aimed to investigate knowledge of obstetric danger signs and associated factors among pregnant women living in Angolela Tera district, northern Ethiopia. The findings of this study will provide important information for the design of an effective intervention programs to enhance women’s knowledge on obstetric danger signs. Moreover, this study serves as a reference material for further research in similar area.

## Main text

### Methods and materials

A community based cross sectional study design was conducted among pregnant women in Angolela Tera district, northern Ethiopia from April 1 to May 15, 2019. Angolela Tera district is found in the northern part of Ethiopia and located at about 113 km from Addis Ababa, the capital city of Ethiopia.

The source population of the study was all pregnant women living in Angolela Tera district and study population was pregnant women who were living in the randomly selected kebeles of Angolela Tera district. The inclusion criteria for this study were pregnant women who had been living in the district for at least 6 months and those who were volunteered to participate in the study. Pregnant women who were seriously ill during data collection and those who were health care workers by occupation were excluded from the study.

The required sample size was determined using single population proportion formula by considering the following assumptions: 95% confidence level, 5% margin of error, proportion of knowledge about obstetric danger signs as 21.9% [14], design effect of 2 and 5% non-response rate. Hence, the total sample size was 552.

Multistage cluster sampling technique was used to select the study subjects. Initially, 1 urban and 10 rural kebeles were selected by lottery method from the total list of 2 urban and 19 rural kebeles of Angolela Tera district. Then, all eligible pregnant women (563) living in the selected kebeles were included in the study. The number of eligible pregnant women obtained during the survey was near to the calculated sample size.

Data were collected through face-to-face interviews using pre-tested structured questionnaire adapted from the Maternal and Neonatal Program of JHPIEGO, an affiliate of John Hopkins University [15]. The questionnaire contained socio-demographic characteristics, reproductive history and knowledge of obstetric danger signs. The questionnaire was translated into local language and the data were collected by trained midwives. To ensure data quality, training was given for data collectors and supervisors for 2 days. Moreover, the questionnaires were pre-tested, the data collection process was supervised and the collected data were checked daily for completeness and consistency.

Knowledge of women about obstetric danger signs were assessed by asking the participants to mention the danger signs that can happen during pregnancy, delivery and post-partum. Then, a woman was considered as knowledgeable when she mentioned at least three key danger signs for each of the three phases (pregnancy, delivery and post-partum) spontaneously [14, 16].

The data were entered into Epi data 3.1 statistical software and then exported to SPSS version 23 for analysis. Descriptive summaries were used to describe socio-demographic characteristics and other independent variables. Additionally, binary logistic regression analysis was performed to identify factors associated with knowledge of obstetric danger signs. All independent variables with p value ≤ 0.25 during the bivariate analyses were further entered to the multivariable binary logistic regression analysis to control for possible confounders.

### Results

#### Socio-demographic characteristics of the participants

A total of 563 pregnant women were included in the study making a response rate of 100%. The mean (standard deviation [SD]) age of the participants was 27 (± 9) years. About 480 (85.3%) study participants were rural residents. By educational status, 297 (52.8%) respondents were illiterate (Table 1).

**Table 1:** Socio-demographic characteristics of pregnant women in Angolela Tera district, Northern Ethiopia, 2019 (n = 563)

Variables	n (%)
Age (in years)	
15–24	212 (37.7)
25–34	217 (38.5)
≥ 35	134 (23.8)
Religion	
Muslim	88 (15.6)
Orthodox	460 (81.7)
Others^a^	15 (2.7)
Marital status	
Single	16 (2.8)
Married	533 (94.7)
Others^b^	14 (2.5)
Educational status	
Illiterate	297 (52.8)
Informal education	56 (9.9)
Formal education	210 (37.3)
Occupation	
Housewife	474 (84.2)
Government employee	24 (5.2)
Private employee	29 (6.4)
Merchant	36 (4.3)
Place of residence	
Rural	480 (85.3)
Urban	83 (14.7)
Household income (ETB per month)	
≤ 500	15 (2.7)
500–1000	74 (13.1)
1001–3000	405 (71.9)
≥ 3001	69 (12.3)

*ETB* Ethiopian birr

^a^Protestant and catholic

^b^Divorced and widowed

#### Obstetric characteristics of the respondents

Among the total respondents, 110 (19.5%) respondents were pregnant for the first time and 65.9% of respondents had at least one antenatal care (ANC) visit for their current pregnancy. About 254 (56.1%) study participants delivered their last child in a health facility and 71.8% of the respondents spent more than 20 min to reach the health institutions (Table 2).

**Table 2:** Obstetric characteristics of respondents in Angolela Tera district, Northern Ethiopia, 2019

Variables	n (%)
Gravidity (n = 563)	
1	110 (19.5)
≥ 2	453 (80.5)
Parity (n = 453)	
1	84 (18.5)
2–4	298 (65.8)
≥ 5	71 (15.7)
History of still birth (n = 453)	
Yes	29 (6.4)
No	424 (93.6)
Antenatal care (ANC) visit for current pregnancy (n = 563)	
No visit	192 (34.1)
One visit	198 (35.2)
Two visit	62 (11.0)
≥ Three visit	111 (19.7)
Place of delivery for the last child (n = 453)	
Home	199 (43.9)
Health institution	254 (56.1)
Have received maternal health education (n = 563)	
Yes	292 (51.9)
No	271 (48.1)
Time taken to reach health facility on foot (n = 563) (min)	
> 20	404 (71.8)
≤ 20	159 (28.2)

#### Knowledge of obstetric danger signs

More than half (60%) of the respondents got information about obstetric danger signs from health care workers. The remaining 34.3% and 5.7% of the participants got information from neighbors and media, respectively. The overall knowledge of study participants about obstetric danger signs was 37.5% (95% CI 33.6–41.5%). About 56.1%, 58.8% and 34.5% of participants were knowledgeable about obstetric danger signs during pregnancy, child birth and postpartum, respectively. Excessive vaginal bleeding was the most frequently mentioned obstetric danger sign in all categories (pregnancy, childbirth and postpartum) (Table 3).

**Table 3:** Knowledge of pregnant women about obstetric danger signs during pregnancy, delivery and postpartum period in Angolela Tera district, Northern Ethiopia, 2019 (n = 563)

Danger signs^a^	Knowledge of danger signs during		
	Pregnancy	Delivery	Postpartum
	n (%)	n (%)	n (%)
Vaginal bleeding	429 (72.6)	371 (65.9)	430 (76.4)
Swollen hands, face and/or feet	72 (12.8)	NA	105 (18.7)
Blurred vision	47 (8.3)	NA	NA
Severe headache	98 (17.4)	NA	NA
Convulsion	55 (9.8)	42 (7.5)	21 (3.7)
No/reduced fetal movement	304 (54)	NA	NA
Water breaks without labor	240 (42.6)	NA	NA
Loss of consciousness	119 (21.1)	64 (11.4)	117 (20.8)
Sever pelvic and abdominal pain	167 (29.7)	NA	NA
Labor lasing > 12 h	NA	365 (64.8)	NA
Retained placenta	NA	281 (49.9)	NA
Baby’s hand/feet comes first	NA	342 (60.7)	NA
Cord comes first of the baby	NA	83 (14.7)	NA
Breathing difficulty	NA	NA	33 (5.9)
Foul smelling lochia	NA	NA	175 (31.1)
High fever	NA	NA	247 (43.9)

*NA* not assessed for that period

^a^Multiple answers possible

#### Factors associated with knowledge of obstetric danger signs

Univariable logistic regression analysis showed that age, gravidity, current antenatal care follow-up, maternal occupation, maternal educational status, source of information for obstetric danger signs, place of residence, time taken to reach health facility on foot and received maternal health education were significantly associated with knowledge of obstetric danger signs at p < 0.05. However, in the multivariable logistic regression analysis attending formal education (AOR; 4.01, 95% CI 2.35–6.75, p; 0.001), urban residence (AOR; 2.01, 95% CI 1.02–5.65, p; 0.013), taken < 20 min to reach health facility (AOR; 5.01, 95% CI 2.76–10.18, p; 0.001), ≥ 2 history of pregnancies (AOR; 2.2, 95% CI 1.2–4.9, p; 0.04) and receiving health education (AOR; 5.31, 95% CI 2.8–10.00, p; 0.002) were found to be significantly associated with knowledge of obstetric danger signs and more likely to have knowledge about obstetric danger signs as compared to their counterparts (Additional file 1: Table S1).

### Discussion

This community based cross sectional study assessed the level of knowledge about obstetric danger signs and associated factors among pregnant women in both urban and rural areas of Angolela Tera district which provides a basic database on the women’s knowledge of the danger signs of obstetric complications.

This study showed that the overall women’s knowledge of obstetric danger signs was 37.5%. This finding is comparable with the studies conducted in Aletawondo, Ethiopia (30.9%) [17], Tanzania (31.3%) [13] and Debre Birhan, Ethiopia (38.6%) [18]. However, the result of this study is lower than studies done in Nepal (66%) [19] and Tigray region, Ethiopia (49.5%) [20]. A lower women’s knowledge of obstetric danger signs was reported in similar cross sectional study conducted in Somali region of Ethiopia [12]. These differences in the findings could be due to socio-cultural differences and variation in the implementation of relevant health intervention programs in the study areas.

The present study indicated that majority of the respondents mentioned vaginal bleeding as danger sign during pregnancy (72.6%), delivery (65.9%), and postpartum period (76.4%). This result is in agreement with previous studies done in different countries [21–23]. The study also revealed that reduced fetal movement (54%), prolonged labor (64.8%) and high fever (43.9%) were other commonly mentioned danger sign during pregnancy, delivery and postpartum period, respectively. While these danger signs are reported as other commonly mentioned danger signs, the figures are lower than a study conducted in Raya Kobo district of Ethiopia [16]. However, while women who can mention the danger signs are correctly classified, other women who might not be able to label those signs when asked would recognize them if they occurred.

In this study, maternal educational status was found to have a significant association with being knowledgeable about obstetric danger signs. Knowledge of obstetric danger signs was higher among women who attended formal education as compared to illiterate women. In line with this finding, other studies [13, 24] showed that an increased maternal educational status was associated with increased women’s knowledge of obstetric danger signs. This might be due to the fact that education provides appropriate information about pregnancy and thus educated women are better informed and make better choices [24]. Moreover, this study indicated that knowledge of obstetric danger signs was more likely to increase among women who received maternal health education during pregnancy as compared to their counterparts. Thus, continuous health education should be provided for pregnant women and also for other relevant parties (traditional birth attendants, community health workers, partners and pregnant women’s relatives) to take prompt measures when the danger signs arise.

Urban residence was also found to have a significant association with being knowledgeable about obstetric danger signs. This finding is consistent with other studies [12, 14, 18, 25]. This could be due to the fact that urban residents are exposed to better health care services and they have better access to relevant health information as compared to rural residents. It was also observed that knowledge of obstetric danger signs was more likely to increase among women who travelled less than 20 min on foot to the health facility from their home. Similar finding was reported in other study [26]. This might be due to easy accessibility of different health facilities and better access to health information for respondents who lived near to health facilities as compared to those living in distant areas.

The result of the present study also revealed that having two or more history of pregnancies increased the likelihood of being knowledgeable about obstetric danger signs, which is in line with previous studies [12, 27]. Women exposed to previous pregnancies could get more information from health professionals due to their previous exposure to health institutions [26].

### Conclusion

In this study, a significant proportion of pregnant women were not knowledgeable about obstetric danger signs during pregnancy, delivery and postpartum. Maternal educational status, residence, time taken to reach health facility on foot, gravidity and maternal health education were found to be independent predictors of knowledge of women about obstetric danger signs. Thus, continuous health education, improving the quality of health information, increasing accessibility of health facilities and appropriate counseling to pregnant mothers could be important to promote the knowledge of pregnant women about obstetric danger signs.

## Limitations

This study has some limitations. First, the data was collected based on self-report of the women pregnancy status and laboratory test for pregnancy was not applied. However, in order to avoid missing of participants with pregnancy, the data was collected in collaboration with the local health extension workers. Second, the cross sectional nature of the study had made it unable to establish the causal relation between knowledge of pregnancy danger signs and explanatory variables.

## Supplementary information

**Additional file 1: Table S1.** Factors associated with knowledge of obstetric danger signs in Angolela Tera district, Northern Ethiopia, 2019 (n = 563). The data showed the result of binary logistic regression analysis which was performed to identify factors associated with knowledge of obstetric danger signs.

## Data Availability

The datasets used and/or analyzed during the current study are available from the corresponding author on reasonable request.

## Abbreviations

ANC: antenatal care
AOR: adjusted odd ratio
CI: confidence interval
COR: crude odd ratio
SD: standard deviation
SPSS: statistical package for social sciences
