# Supplementary material for: Knowledge of obstetric danger signs and its associated factors among pregnant women in Angolela Tera District, Northern Ethiopia
Source: BMC Res Notes. 2019 Sep 23;12:606. doi: 10.1186/s13104-019-4639-8 (PMC6755683; doi:10.1186/s13104-019-4639-8)
Supplement: Supplementary file 1 — Additional file 1: Table S1. Factors associated with knowledge of obstetric danger signs in Angolela Tera district, Northern Ethiopia, 2019 (n = 563). The data showed the result of binary logistic regression analysis which was performed to identify factors associated with knowledge of obstetric danger signs. [file 13104_2019_4639_MOESM1_ESM.docx]

**Table S1:** Factors associated with knowledge of obstetric danger signs in Angolela Tera district, Northern Ethiopia, 2019 (n = 563)

| **Variables Knowledge of ODS**  **COR (95% CI) AOR (95% CI) p value**  **No Yes** |
| --- |
| **Age (in years)**  15−24 156 56 1.00 1.00  25 – 34 133 84 1.76 (1.17,2.65) 0.98 (0.53,1.8) 0.96  ≥ 35 63 71 3.14 (1.99,4.96) 1.2 (0.59, 2.44) 0.61  **Educational status**  Illiterate 237 58 1.00 1.00  Informal education 38 16 1.72 (0.9, 3.3) 1.68 (0.78, 3.6) 0.2  Formal education 77 137 7.27 (0.8, 10.87) 4.01(2.35, 6.75) **0.001**  **Occupation**  Housewife 321 153 1.00 1.00  Government employee 12 12 4.7 (2.07,10.48) 2.36 (0.85,6.57) 0.16  Private employee 9 20 5.5 (2.57,11.60) 3.07 (0.9,6.05) 0.72  Merchant 10 26 2.1 (0.92,4.78) 0.67 (0.25,1.8) 0.43  **Place of residence**  Rural 322 170 1.00 1.00  Urban 30 41 2.59 (1.43,4.3) 2.01(1.02,5.65) **0.013**  **Travel time to**  **health center (on foot)**  > 20 minutes 303 101 1.00 1.00  ≤ 20 minutes 49 110 6.74 (4.49,10.1) 5.01 (2.76,10.18) **0.001**  **Source of information**  Neighbors 163 30 1.00 1.00  Health care workers 165 173 5.7 (0.65, 8.88) 1.74 (0.96, 3.15) 0.071  Media (radio, television) 24 8 1.8 (0.74,4.4) 0.73 (0.23,2.3) 0.93  **Gravidity**  1 90 20 1.00 1.00  ≥ 2 262 191 3.3 (1.95,5.5) 2.20 (1.2, 4.9) **0.04**  **Received health education**  Yes 114 178 11.26 (7.3,17.4) 5.31 (2.8, 10.0) **0.002**  No 238 33 1.00 1.00  **Current antenatal care visit**  Yes 186 185 6.35 (4.01,10.07) 1.02 (0.52, 2.01) 0.96  No 166 26 1.00 1.00 |

COR; Crude odds ratio, AOR; Adjusted odds ratio, CI; Confidence interval, ODS; Obstetric danger signs, 1.00; reference group, Numerical data in bold results are significant.
